# Computed Tomography-Based Texture Features for the Risk Stratification of Portal Hypertension and Prediction of Survival in Patients With Cirrhosis: A Preliminary Study

**DOI:** 10.3389/fmed.2022.863596

**Published:** 2022-04-01

**Authors:** Shang Wan, Yi Wei, Xin Zhang, Caiwei Yang, Fubi Hu, Bin Song

**Affiliations:** ^1^Department of Radiology, West China Hospital, Sichuan University, Chengdu, China; ^2^Pharmaceutical Diagnostics, GE Healthcare, Beijing, China; ^3^Department of Radiology, First Affiliated Hospital of Chengdu Medical College, Chengdu, China; ^4^Department of Radiology, Sanya People’s Hospital, Sanya, China

**Keywords:** risk stratification, survival, computed tomography, texture features, portal hypertension

## Abstract

**Objective:**

Clinical evidence suggests that the risk stratification of portal hypertension (PH) plays a vital role in disease progression and patient outcomes. However, the gold standard for stratifying PH [portal vein pressure (PVP) measurement] is invasive and therefore not suitable for routine clinical practice. This study is aimed to stratify PH and predict patient outcomes using liver or spleen texture features based on computed tomography (CT) images non-invasively.

**Methods:**

A total of 114 patients with PH were included in this retrospective study and divided into high-risk PH (PVP ≥ 20 mm Hg, *n* = 57) or low-risk PH (PVP < 20 mm Hg, *n* = 57), a progression-free survival (PFS) group (*n* = 14), or a non-PFS group (*n* = 51) based on patients with rebleeding or death after the transjugular intrahepatic portosystemic shunt (TIPS) procedure. All patients underwent contrast-enhanced CT, and the laboratory data were recorded. Texture features of the liver or spleen were obtained by a manual drawing of the region of interest (ROI) and were performed in the portal venous phase. Logistic regression analysis was applied to select the significant features related to high-risk PH, and PFS-related features were determined by the Cox proportional hazards model and Kaplan-Meier analysis. Receiver operating characteristic (ROC) curves were used to test the diagnostic capacity of each feature.

**Results:**

Five texture features (one first-order feature from the liver and four wavelet features from the spleen) and the international normalized ratio (INR) were identified as statistically significant for stratifying PH (*p* < 0.05). The best performance was achieved by the spleen-derived feature of wavelet.LLH_ngtdm_Busyness, with an AUC of 0.72. The only log.sigma.3.0.mm.3D_firstorder_RobustMeanAbsoluteDeviation feature from the liver was associated with PFS with a C-index of 0.72 (95% CI 0.566–0.885), which could stratify patients with PH into high- or low-risk groups. The 1-, 2-, and 3-year survival probabilities were 66.7, 50, and 33.3% for the high-risk group and 93.2, 91.5, and 84.4% for the low-risk group, respectively (*p* < 0.05).

**Conclusion:**

CT-based texture features from the liver or spleen may have the potential to stratify PH and predict patient survival.

## Introduction

Portal hypertension (PH) is the initial and main consequence of cirrhosis and is responsible for the majority of its complications (1), which, by definition, is an increase in the pressure in the portal vein and its territory (2). The direct measurement of portal vein pressure (PVP) is the most accurate technique for reflexing PH, but it is extremely invasive (2). Thus, the indirect and less invasive measurement of the hepatic venous pressure gradient (HVPG), widely accepted as the PVP equivalent, has been applied in clinical practice (2–4).

In recent years, clinically significant portal hypertension (CSPH) has been recognized in patients with liver cirrhosis and is defined by an HVPG of at least 10 mm Hg, which is associated with an increased risk of variceal bleeding, hepatic encephalopathy (HE), post-surgical decompensation (5), and hepatocellular carcinoma (HCC) (6). Patients at this stage may have different prognoses based on the level of HVPG (7); notably, an HVPG of at least 20 mm Hg is considered a strong predictor of early rebleeding and death (8, 9), which would put patients at higher risk of decompensation and poor clinical outcome. The findings of these studies revealed the clinical significance of identifying severe PH. Previous studies also indicated that recurrent variceal bleeding occurs in 60% of patients after variceal rupture, if untreated, usually within 1–2 years of index hemorrhage (1, 10). Herein, the risk stratification of PH and individualizing care for patients are warranted in clinical decision-making.

Despite the crucial role of PVP or HVPG measurements for the assessment and prognostic evaluation of PH (11), the invasive nature and high-cost effectiveness of these techniques have limited their clinical application as ideal surveillance tools for monitoring disease progression (12). Currently, liver stiffness (LS) by transient elastography (TE; Fibro-Scan) is recognized as the backbone of the non-invasive diagnosis of PH (1, 13); however, controversy still exists regarding its application in patients with obesity, non-alcoholic fatty liver disease, or severe ascites (1). In the past few years, imaging modalities have shown potential in the assessment of PH as non-invasive and effective procedures (12). The literature has demonstrated that computed tomography (CT) has shown promising results for diagnosing PH based on morphological measurements or computational algorithms (9, 14, 15), however, non-invasive stratification of PH on images has not been specified and remains challenging.

Most patients with PH asking for medical help present overt clinical manifestations, such as varices or variceal hemorrhage (12), which, by definition, with CSPH. Abraldes et al. indicated that an HVPG ≥ 20 mm Hg is an independent factor that predicts failure to control bleeding in patients with PH (16), and another study demonstrated that HVPG is the only variable associated with patient outcome and that an HVPG ≥ 20 mm Hg predicts poor evolution when compared with HVPG < 20 mm Hg, specifically, longer intensive care unit stay, longer hospital stay, and greater transfusion requirements. Thus, stratifying PH and further predicting patients’ clinical outcomes with non-invasive tests are urgently needed for patient management (1, 7).

Texture analysis can non-invasively extract digital information from images that naked eyes cannot with a high throughput and can thus explore more characteristics and provide more quantitative information from images (17). This imaging-based technique has been applied to tumor characterization, differential diagnosis, and prediction of prognosis (17–20). A landmark report indicated that the radiomics signature extracted from CT could achieve significant clinical benefits in the detection of CSPH (14). Another study found that CT-based radiomics features may predict PVP (21); however, the specified stratification of PH has not yet been investigated. To the best of our knowledge, there is still a lack of reports on CT-based texture features for the stratification of PH and the prediction of survival conditions in patients with PH.

In this study, we aimed to assess whether CT-based liver or spleen texture signatures could be used to predict high-risk PH and patients’ long-term clinical outcomes.

## Materials and Methods

### Patients

**FIGURE 1:**
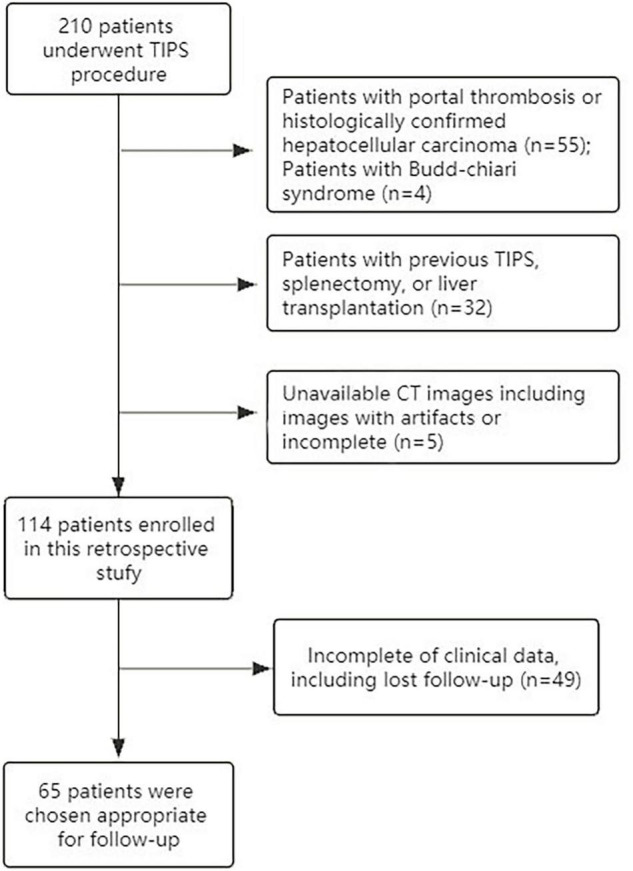
The flowchart of patient enrollment.

This retrospective study was approved by the West China Hospital Ethics Committee and had a waiver of patients’ written informed consent. This study was conducted following the Declaration of Helsinki. From January 2016 to October 2020, patients with PH admitted to our medical center for a transjugular intrahepatic portosystemic shunt (TIPS) procedure were eligible for study participation. The inclusion criteria were as follows: (1) patients who were diagnosed with liver cirrhosis; (2) patients with available intraoperative direct measurements of PVP and abdominal contrast-enhanced CT scans; and (3) adult patients (age ≥ 18 years). The exclusion criteria were as follows: (1) patients who previously underwent one of the following surgical procedures: TIPS, splenectomy, partial splenic embolization, balloon-occluded retrograde, transvenous obliteration, or liver transplantation; (2) patients with portal thrombosis or histologically confirmed HCC; and (3) patients with non-sinusoidal PH (e.g., hepatic cavernoma, Budd-Chiari syndrome). All patients received the TIPS procedure with direct PVP measurement during this hospitalization and underwent contrast-enhanced CT within 4 weeks prior to the TIPS procedure. The patients’ laboratory assessments were also recorded, and the patients were divided into a high-risk PH group (PVP ≥ 20 mm Hg) and a low-risk PH group (PVP < 20 mm Hg) according to the PVP levels. The flowchart of patient enrollment is shown in Figure 1.

### Transjugular Intrahepatic Portosystemic Shunt Procedure

The TIPS procedure was performed using a previously described standard process (22). The jugular vein was accessed and a TIPS set (Cook Medical Co., Bloomington, IN, United States) was introduced into the right hepatic vein. The metal cannula was bent by the operator according to the anatomical relationship between the hepatic vein and the targeted puncture site along the portal vein branch. A 3D roadmap was used for portal vein puncture guidance, and access to the portal vein was confirmed by injecting the contrast using a 5-ml syringe under fluoroscopy. Subsequently, direct portography was performed, and PVP measurements were made. The intrahepatic parenchymal tract was then dilated with an 8-mm balloon (Powerflex; Cordis, Roden, Netherlands) and an 8-mm stent graft (Fluency; C.R. Bard, Murray Hill, NJ, United States) was placed. The direct PVP was measured again, and the targeted threshold after stent deployment was 12 mm Hg (23, 24).

### Computed Tomography Image Acquisition

The investigated individuals underwent contrast-enhanced CT imaging with one of the following systems: Sensation 64 CT (Siemens), Sensation 16 CT (Siemens), or 64 LightSpeed VCT (GE Healthcare). Triple-phase CT examinations were conducted, i.e., non-enhanced, arterial, and portal vein phases. Abdominal scouts were acquired from the dome of the diaphragm to the iliac crests. The arterial phase of the same region was started at approximately 20–30 s after contrast agent administration and was followed by the portal phase (30–40 s). The reconstructions were conducted on a GE Advantage Windows 3D workstation (GE Healthcare, Waukesha, WI, United States), and the reconstitution thickness was set at 1–2 mm. The detailed scanning parameters are listed as follows: tube voltage, 120 or 100 kVp; tube current, 150–600 mA; slice thickness, 1.25 mm; and pitch, 1.375. All patients received an intravenous, non-ionic contrast agent (iodine concentration, 370 mg/ml; volume, 1.5–2.0 ml/kg of body weight; contrast type, Omnipaque 300, GE Healthcare, Ireland) at a rate of 3–5 ml/s. A volume of 20 ml saline was injected after the injection of the contrast.

### Follow-Up

Patients were consistently followed up after the TIPS procedure by periodic re-examinations of CT scans in the outpatient clinics at intervals of 3–6 months or by telephone verification. The time of disease-specific progression (rebleeding) or death was recorded, and patients were censored on October 30, 2021. Patients for follow-up were divided into a progression-free survival (PFS) group or a non-PFS group based on patients with rebleeding or death after the TIPS procedure.

### Texture Feature Extraction

**FIGURE 2:**
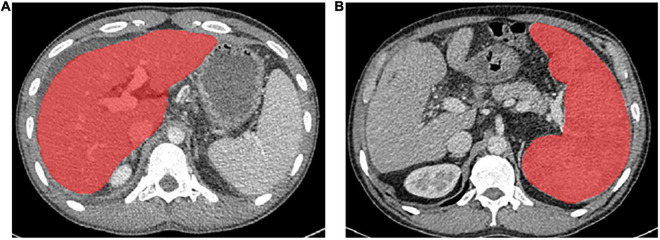
The delineation of the region of interest of the liver **(A)** and spleen **(B)** on CT images.

Portal venous phase CT images were used for texture feature extraction (14, 25). Regions of interest (ROIs) were drawn around the liver at the porta hepatis level and around the spleen at the splenic hilum level using ITK-SNAP 3⋅6 (ITK- SNAP 3⋅X TEAM) (14). Then, Artificial Intelligence Kit software (A.K. software; GE Healthcare, Life Sciences, Beijing, China) was used to extract feature parameters for each ROI, which was based on the image biomarker standardization initiative (IBSI). Figure 2 shows the delineation of the ROI of the liver and spleen. Before feature extraction, image normalization was performed by remapping the histogram to fit μ ± 3σ: (μ, average grayscale within ROI; σ, grayscale SD) (26). Texture features were also extracted from images conducted with the Laplacian of Gaussian filter (Log) and wavelet filter. All scans were analyzed by two senior residents independently (CWY, 5 years of experience in abdominal imaging analysis, and YW, 8 years of experience in abdominal imaging analysis) and were supervised by a senior radiologist (FY, 13 years of experience) to handle the non-consensus.

### Texture Analysis and Statistical Analysis

A total of 1,037 radiomics features were calculated for original images and filtered images from liver or spleen segmentation that include first-order features of 18 intensity statistics and 14 3D shape features, 24 gray-level co-occurrence matrix (GLCM), 16 gray-level size zone matrix (GLSZM), 16 gray-level run length matrix (GLRLM), 5 neighboring gray-tone difference matrix (NGTDM), and 14 gray-level dependence matrix (GLDM) features and features with two filters that include 744 wavelet features and 186 LoG filtered features.

The intraclass correlation coefficient (ICC) was considered to evaluate the interobserver agreement, and ICC values of > 0.85 represent an almost perfect agreement between observers. The Mann-Whitney *U*-test was used to compare continuous variables, and the chi-square test was used to compare categorical variables. Univariate and multivariate logistic regression analyses were used to screen the independent risk factors for discriminating the high-risk or low-risk PH group. Univariate analyses were performed first, and only parameters found to have statistical significance were used for further stepwise multivariate logistic regression.

The diagnostic performance of each texture feature for discriminating the high-risk or low-risk PH group was quantified by the receiver operating characteristic (ROC) curve and the area under the curve (AUC), and the accuracies, sensitivities, and specificities were also calculated. Additionally, univariate analyses with Cox proportional hazards regression identified the predictors of disease progression of variceal bleeding recurrence and death. The Cox proportional hazards model was used to assess the PFS-associated texture features that predicted the probabilities of 1-, 2-, and 3-year PFS in the followed up patients. The risk probability of followed up patients was stratified into high-risk and low-risk groups using the optimal cutoff point determined by X-tile software (27). Survival curves were generated with the Kaplan-Meier method and compared by a 2-sided log-rank test. The C-index was used to determine the diagnostic capabilities of risk factors associated with PFS.

Categorical variables are reported as frequencies and proportions. Continuous variables are reported as the means (SD) and medians (interquartile ranges, IQR). Statistical analysis was performed using R software (version 3.5.3). A values of *p* of less than 0.05 was defined as significant in two-tailed analyses.

## Results

### Patient Characteristics

The characteristics of the patients are summarized in Table 1. Out of 114 patients included, there were 57 cases in the high-risk PH group (PVP ≥ 20 mm Hg) and 57 cases in the low-risk PH group (PVP < 20 mm Hg). A total of 65 patients were finally followed that include 14 cases in the PFS group and 51 cases in the non-PFS group. In the low- and high-risk PH groups, the value of the international normalized ratio (INR) was found to be statistically significant between these two groups (*p* < 0.05), except that the remaining clinical parameters were not statistically significant between the two groups (Table 1).

**TABLE 1:** Demographics and clinical characteristics of the study population.

Variables	*N*	Low-risk PH	High-risk PH	Statistics	*P*
Age, years*^a^*	114	48.64 ± 11.45	52.04 ± 8.87	−1.711	0.091
**Gender** *^b^*				1.671	0.196
Male	78	29 (61.70%)	49 (73.13%)		
Female	36	18 (38.30%)	18 (26.87%)		
**Etiology** *^b^*				−0.066	0.947
Post-hepatic cirrhosis	73	32 (68.09%)	41 (61.19%)		
Alcoholic cirrhosis	14	2 (4.26%)	12 (17.91%)		
Combined cirrhosis	12	4 (8.51%)	8 (11.94%)		
Primary biliary cirrhosis	7	3 (6.38%)	4 (5.97%)		
Others	8	6 (12.77%)	2 (2.99%)		
**Child**–**Pugh class***^b^*				−0.253	0.8
Child–Pugh class A	35	15 (31.91%)	20 (29.85%)		
Child–Pugh class B	61	25 (53.19%)	36 (53.73%)		
Child–Pugh class C	18	7 (14.89%)	11 (16.42%)		
**PVP** (mm Hg)*^c^*	114	17.00 (15.20, 18.00)	22.00 (21.00, 27.00)	−8.805	<0.001
**EVB history** *^b^*				1.131	0.288
Absent	17	9 (19.15%)	8 (11.94%)		
Present	97	38 (80.85%)	59 (88.06%)		
**Ascites** *^b^*				0.77	0.38
Absent	20	10 (21.28%)	10 (14.93%)		
Present	94	37 (78.72%)	57 (85.07%)		
**Hypersplenism** *^b^*				0.337	0.561
Absent	88	35 (74.47%)	53 (79.10%)		
Present	26	12 (25.53%)	14 (20.90%)		
**Hepatic encephalopathy** *^b^*				2.433	0.119
Absent	105	46 (97.87%)	59 (88.06%)		
Present	9	1 (2.13%)	8 (11.94%)		
Total bilirubin*^c^*	114	21.50 (13.30, 30.72)	26.30 (16.34, 32.46)	−1.361	0.173
Albumin*^a^*	114	34.40 ± 6.50	33.09 ± 6.12	1.096	0.275
Globulin*^c^*	114	27.60 (23.68, 31.82)	26.90(22.62, 32.06)	0.653	0.514
ALT*^c^*	114	21.00 (13.20, 31.80)	21.00 (14.00, 43.40)	−0.458	0.647
AST*^c^*	114	32.00 (22.00, 43.80)	31.00 (21.20, 55.60)	−0.622	0.534
INR*^c^*	114	1.24 (1.15, 1.39)	1.35 (1.23, 1.48)	−2.331	0.02
PLT*^c^*	114	62.00 (51.20, 89.20)	63.00 (40.20, 100.40)	0.636	0.525

*PH, portal hypertension; PVP, portal vein pressure; EVB, esophageal variceal bleeding; ALT, alanine aminotransferase; AST, aspartate aminotransferase; INR, international normalized ratio; PLT, platelet count.*

*^a^Data were compared by using Student’s t-test and are presented as the means ± deviation.*

*^b^Data were compared using chi-square test and are presented as numbers (%).*

*^c^Data were compared using the Mann-Whitney test and are presented as medians (interquartile range).*

### Clinical Variables and Texture Features for Portal Hypertension Stratification

Texture features that had greater ICCs considering a threshold of 0.85 were robust and adopted for later analysis. Of all the clinical factors or CT-based texture features, six significant features, i.e., 1 clinical variable [INR, odds ratio (OR) 7.76, 95% confidence interval (CI) 1.14–52.88], and 5 texture features, were identified as independent predictors by univariate analysis. Out of the 5 CT-based texture features, log.sigma.3.0.mm.3D_firstorder_RobustMeanAbsoluteDeviation was identified from the liver (OR: 0.71, 95% CI: 0.54–0.94), and wavelet.LLH_ngtdm_Busyness (OR 3.74, 95% CI 1.28–10.9), wavelet.HLL_glrlm_RunLengthNonUniformity (OR 2.08, 95% CI 1.1–3.95), avelet.HLH_glcm_MCC (OR 0.57, 95% CI 0.34–0.95), and wavelet.LLL_glrlm_RunLengthNonUniformity (OR 2.1, 95% CI 1.09–4.05) were identified in the spleen (Table 2). Clinical variable of INR and spleen-derived feature of wavelet.LLH_ngtdm_Busyness showed the most significant association with the high-risk PH group (OR 7.76, 95% CI 1.14–52.88, *p* = 0.036 vs. OR 3.74, 95% CI 1.28–10.9, *p* = 0.016).

**TABLE 2:** Univariate analysis for stratifying portal hypertension.

Variables	Prediction of high-risk PH	
	OR (95% CI)	*P*
log.sigma.3.0.mm.3D_firstorder_RobustMeanAbsoluteDeviation	0.71 (0.54–0.94)	0.017
wavelet.LLH_ngtdm_Busyness	3.74 (1.28–10.9)	0.016
wavelet.HLL_glrlm_RunLengthNonUniformity	2.08 (1.1–3.95)	0.025
wavelet.HLH_glcm_MCC	0.57 (0.34–0.95)	0.03
wavelet.LLL_glrlm_RunLengthNonUniformity	2.1 (1.09–4.05)	
INR	7.76 (1.14–52.88)	0.036

*PH, portal hypertension; OR, odds ratio; CI: confidence interval; INR, international normalized ratio.*

**FIGURE 3:**
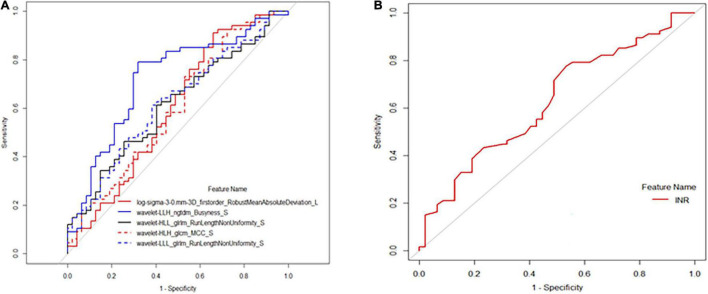
Receiver operating characteristic (ROC) analysis of selected texture. features and international normalized ratio (INR) for predicting high-risk portal hypertension (PH). **(A)** The ROC curve calculated by texture features of log.sigma.3.0.mm.3D_firstorder_RobustMeanAbsoluteDeviation, wavelet.LLH_ngtdm_Busyness, wavelet.HLL_glrlm_RunLengthNonUniformity, wavelet.HLH_glcm_MC, and wavelet.LLL_glrlm_RunLengthNonUniformity. **(B)** The ROC curve of INR for predicting high-risk PH.

Receiver operating characteristic analysis showed that the above texture features had moderate capabilities to distinguish between the high- and low-risk PH groups, of which the best performance was achieved by the spleen-derived feature of wavelet.LLH_ngtdm_Busyness, with an AUC of 0.72, an accuracy of 0.746, a specificity of 0.681, and a sensitivity of 0.791 when using a cutoff value of 0.517 (Table 3 and Figure 3). The clinical feature of INR also showed a moderate performance for stratifying PH, with an AUC of 0.629, an accuracy of 0.649, a specificity of 0.468, and a sensitivity of 0.776 when using a cutoff value of 0.528 (Figure 3).

**TABLE 3:** The performance of texture features and INR for stratifying portal hypertension.

Variables	AUC (95% CI)	Accuracy	Specificity	Sensitivity	Cutoff
log.sigma.3.0.mm.3D_firstorder_RobustMeanAbsoluteDeviation	0.605 (0.49–0.710)	0.658	0.333	0.899	0.493
wavelet.LLH_ngtdm_Busyness	0.72 (0.622–0.817)	0.746	0.681	0.791	0.517
wavelet.HLL_glrlm_RunLengthNonUniformity	0.594 (0.509–0.717)	0.55	0.843	0.333	0.618
wavelet.HLH_glcm_MCC	0.593 (0.489–0.705)	0.65	0.275	0.928	0.5
wavelet.LLL_glrlm_RunLengthNonUniformity	0.604 (0.518–0.726)	0.592	0.588	0.594	0.553
INR	0.629 (0.525–0.733)	0.649	0.468	0.776	0.528

*AUC, the area under the ROC curve; CI, confidence interval; INR, international normalized ratio.*

### Texture Features for Progression-Free Survival

**FIGURE 4:**
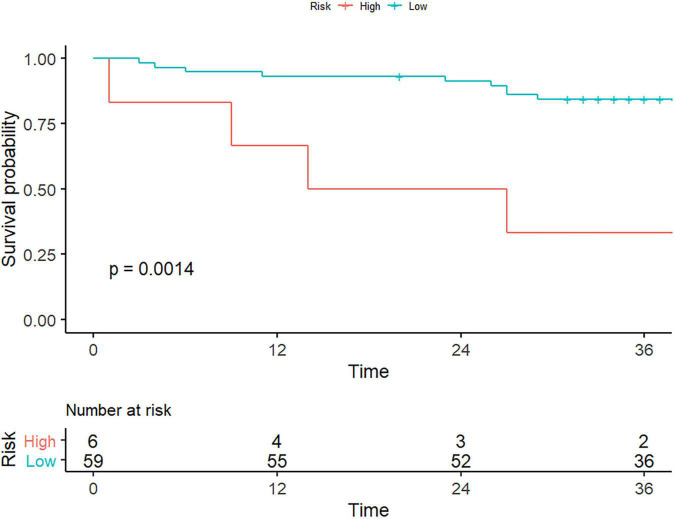
Texture features for the evaluation of patient survival. As the picture depicts, only the liver-derived feature of log.sigma.3.0.mm. 3D_firstorder_RobustMeanAbsoluteDeviation could stratify patients with portal hypertension into high- or low-risk group (log-rank test, *p* < 0.05).

As of October 30, 2021, a total of 65 of 114 (57.0%) patients had completed the PFS follow-up, the overall recurrence rate of bleeding was 12.3% (8/65), and the overall death rate was 9.2% (6/65). Table 4 shows the results of univariate Cox proportional hazard regression analysis for PFS, of which only log.sigma.3.0.mm.3D_firstorder_RobustMeanAbsoluteDeviation had a statistically significant difference for the PFS stratification and could divide the followed up patients into high- or low-risk groups (log-rank test, *p* < 0.05; Figure 4). It was lower in the high-risk group (medium 3.839; IQR 3.465–4.027) than in the low-risk group (medium 5.868; IQR 5.166–6.942) [hazard ratio (HR) 0.529, 95% CI 0.322–0.869, *p* = 0.012], and the remaining texture features were not found to be associated with PFS. The feature of log.sigma.3.0.mm.3D_firstorder_RobustMeanAbsoluteDeviation presented a moderate prognostic performance for predicting the high-risk group with a C-index of 0.72 (95% CI 0.566–0.885) when using a cutoff value of 4.15. We also evaluated clinical characteristics for survival using univariate Cox proportional hazard regression. We found that the variables of hypersplenism and HE had statistical significance for survival analysis for PFS (*p* < 0.05; Supplementary Table 1), with HRs of 3.80 (95% CI 1.31–10.99) and 4.27 (95% CI 1.33–13.64), respectively. Supplementary Figures 1, 2 show the survival curves of hypersplenism and HE, respectively. Clinical manifestations of hypersplenism presented a C-index of 0.643 (95% CI 0.514–0.772), and HE presented a C-index of 0.614 (95% CI 0.494–0.734).

**TABLE 4:** Univariate Cox proportional hazard regression for survival.

Variables	Survival analysis for PFS	
	HR (95% CI)	*P*
log.sigma.3.0.mm.3D_firstorder_RobustMeanAbsoluteDeviation	0.529 (0.322–0.869)	0.012
wavelet.LLH_ngtdm_Busyness	0.727 (0.266–1.991)	0.535
wavelet.HLL_glrlm_RunLengthNonUniformity	1.053 (0.671–1.653)	0.821
wavelet.HLH_glcm_MCC	1.352 (0.775–2.361)	0.288
wavelet.LLL_glrlm_RunLengthNonUniformity	1.03 (0.651–1.63)	0.9

*HR, hazard ratio; CI, confidence interval; PFS, progression-free survival.*

**FIGURE 5:**
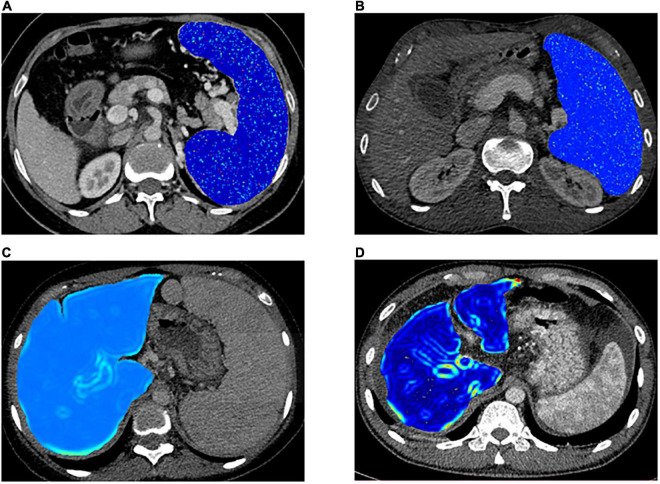
Representative cases for stratifying portal hypertension (PH) and patients’ survival condition. **(A,B)** Visualized spleen-derived texture feature of wavelet.LLH_ngtdm_Busyness in high-risk PH patients [portal vein pressure (PVP) ≥ 20 mm Hg] and low-risk PH patients (PVP < 20 mm Hg) respectively. **(C,D)** Visualized liver-derived texture feature of log.sigma.3.0.mm.3D_firstorder_RobustMeanAbsoluteDeviation in the high-risk patients and low-risk patients, respectively.

The median survival time was 20.5 (IQR 10.25–39.75) months for high-risk patients and 37 (IQR 32.5–41.5) months for low-risk patients. The 1-, 2-, and 3-year survival probabilities were 66.7, 50, and 33.3% for the high-risk group and 93.2, 91.5, and 84.4% for the low-risk group, respectively (log-rank test, *p* = 0.0014). Representative cases were given to show the discriminative performance of the features for stratifying PH and predicting PFS (Figure 5).

## Discussion

Non-invasive stratification of PH and prediction of high-risk PH in patients with cirrhosis have been highlighted in recent years due to the lack of widespread application of invasive PVP or HVPG measurements. In this study, we assessed the texture features based on CT and clinical data non-invasively for predicting high-risk PH patients. In addition, we evaluated patient outcomes using the extracted features, with the aim of aiding clinical decision-making.

Our results suggested that texture features from the liver or spleen were significantly different between the high-risk PH and low-risk PH groups in cirrhotic patients that include log.sigma.3.0.mm.3D_firstorder_RobustMeanAbsoluteDeviation from the liver, wavelet.LLH_ngtdm_Busyness, wavelet.HLL_glrlm_RunLengthNonUniformity, wavelet.HLH_glcm_ MCC, and wavelet.LLL_glrlm_RunLengthNonUniformity from the spleen. Out of these features, the feature of wavelet.LLH_ngtdm_Busyness from the spleen demonstrated the best diagnostic performance, with an AUC of 0.72. Furthermore, we found that only the feature of log.sigma.3.0.mm.3D_firstorder_RobustMeanAbsoluteDeviation was associated with patient outcomes, and it also showed a moderate prognostic capability for discriminating the high-risk group from the low-risk group, with a C-index of 0.726 based on a cutoff value of 0.415.

A previous study demonstrated that the non-invasive radiomics signature based on a machine-learning method, which they termed *r*HVPG, could accurately facilitate the diagnosis of PH in patients with cirrhosis (14). Their findings underlined the significance of the detection of CSPH in clinical treatment and inspired more investigation using the advanced machine-learning algorithm for the evaluation of PH. However, current guidelines indicate that different levels of portal pressure are a strong predictor for patient outcomes (1). As mentioned previously, an HVPG ≥ 20 mm Hg predicts poor patient long-term survival and a higher incidence of rebleeding (8, 16); thus, stratification of PH and identification of severe PH should be more emphasized. Therefore, we conducted a further investigation based on a previous report (14), and we evaluated the performance of the texture signature from the machine-learning method for the stratification of PH.

We found that out of 5 texture features associated with high-risk PH, four were derived from the spleen, which might refer to previous literature. They found that non-invasive spleen-related parameters have the potential to predict the grade of PH and the presence of varices (28–30). For example, spleen stiffness measurement by Fibro-Scan has been found to be more closely related to PH than LS measurement (31, 32). The following reason might explain the spleen-related finding of the present study. We all know that patients with severe PH generally present spleen enlargement in the natural history of disease progression. It is relevant to note that splenomegaly in cirrhosis is characterized by enlargement and hyperactivation of the splenic lymphoid tissue and increased angiogenesis and fibrogenesis, in addition to passive congestion due to increased portal pressure (33, 34). Briefly, the pathogenetic changes leading to spleen enlargement can be reflected in the spleen tissue that includes the outer splenic morphological features and the inner compartment; thus, the measurement of spleen stiffness could reveal the physical property of spleen tissue consequent to the hyperactivation condition of PH, by which satisfactory results were obtained to be closely correlated with the degree of PH, at least not inferior to that of LS, particularly, in a more advanced stage of PH (32); therefore, spleen stiffness showed a close relationship with PH. Likely, as an advanced imaging-based technique, texture analysis can extract more valuable data of the tissue component that traditional methods cannot detect, partially, such as spleen stiffness measurement (17), it might be able to reveal more inner pathologic characteristics of the spleen and can thus have the ability to correlate with PH, especially in a more advanced stage of PH as mentioned previously, for example, the stage of the high-risk PH.

Tseng et al. indicated that a radiomics model based on the spleen signature can yield superior performance for predicting portal pressure when compared with the model of the liver signature (AUC 0.832 vs. 0.789, respectively) (21), which highlighted the spleen-derived signature on images. However, they only evaluated the association between portal pressure and the radiomics model and failed to further investigate the risk stratification of PH. In this study, similar results were observed in the diagnostic performance of spleen-derived texture features (AUC, wavelet.LLH_ngtdm_Busyness of 0.72 from spleen vs. log.sigma.3.0.mm.3D_firstorder_RobustMeanAbsoluteDeviation of 0.605 from liver). The spleen-derived texture outperformed that of the liver and seemed more suitable to evaluate PH. We speculated that the splenic-dominated result may be associated with the complex vascular branch, particularly, the opening of portosystemic shunts in the late stage of PH, which may have significant implications on the liver tissue (35, 36). As a result, texture features from the liver might not be able to reflect the complex hemodynamic changes of severe PH and may not correlate well with severe PH. However, as a relatively isolated organ, the spleen may not be influenced as much as the liver by the collateral circulation in the late stage of PH (37, 38); thus, the spleen-derived features seem more stable and reliable. The findings of this study suggest the potential of splenic texture features for the prediction of high-risk PH; however, due to the lack of relevant literature regarding the stratification of PH and the relatively limited diagnostic capability, the results of this study should be interpreted cautiously and need to be corroborated in further prospective studies.

The clinical value of INR in the evaluation of liver cirrhosis and the prognostic condition of patients still remains controversial (1, 39). Malinchoc et al. have previously reported that INR for prothrombin time (PT) could be used as a predictor for survival conditions in patients with liver cirrhosis undergoing the TIPS procedure (40). Zhang et al. reported that the INR and PT in the bleeding group were higher than those in the non-bleeding group in patients with cirrhosis (41). They indicated that the liver is an important site for coagulation factor synthesis, and INR represents the deficiency in procoagulant proteins in cirrhosis (42). The changes in PT and INR can accurately reflect the degree of liver function impairment, and a longer PT or INR usually suggests a worse liver function (41, 43). However, the association between INR and portal pressure has not been described, and our results suggested a higher level of INR in the high-risk PH group than in the low-risk PH group (medium 1.35 vs. 1.24), with an OR of 7.76. As we mentioned above, patients with a higher INR are often accompanied by poor liver function; theoretically, patients with poor liver function [Child-Turcotte-Pugh (CTP) B or C] are usually decompensated or at an advanced stage of PH (1), given that a higher INR value may be associated with severe PH, such as high-risk PH. Regarding this, the findings of this study with INR may provide valuable information for clinicians for the stratification of PH.

To the best of our knowledge, this is the first report that predicts patient outcomes consequent to PH using texture signatures from the liver or spleen. Based on the significant features for diagnosing high-risk PH patients, we found that only the feature of log.sigma.3.0.mm.3D_firstorder_RobustMeanAbsoluteDeviation was associated with patient outcomes. A previous study applied the radiomics technique in the assessment of portal pressure along with the outcome (21); however, they only evaluated patients’ outcome of variceal recurrence after initial endoscopic therapy (suggesting a high portal pressure) (44), by which the association between the portal pressure and the radiomics was obtained. Unlike the previous study, in this study, we evaluated patients’ survival condition more directly by collecting the data of recurrence of bleeding or death, which can be more clinically relevant and may provide more helpful information for disease progression, and we found that the feature can yield a moderate capability for discriminating the high-risk group from the low-risk group when using a cutoff value of 4.15 in this preliminary study, obtaining a C-index of 0.726. We know that texture signatures can quantify image features by extracting the distribution and relation of pixel or voxel grayscale in images (17), and the 3D feature (log.sigma.3.0.mm.3D_firstorder_RobustMeanAbsoluteDeviation) indicates the rate of intensity change of the images. In this study, we found a lower value of that in the high-risk group than that in the low-risk group (medium 3.839 vs. 5.868), suggesting a higher homogeneity of images in the high-risk group. Furthermore, the only significant feature was derived from the liver; we assumed that the difference between the high- and low-risk groups may correlate with the inner alteration of liver tissue consequent to cirrhosis. Additionally, we found that clinical variables of hypersplenism and HE were also associated with PFS, and this finding is consistent with the evolution of cirrhosis and PH (1). Since PH is not an isolated complication, it should be considered in the context of advances in the staging of cirrhosis and in the context of other complications of cirrhosis (1, 45). Whether these variables can be used as independent prognostic factors for PFS should be validated with more studies.

Several limitations of our study should be noted. First, due to the retrospective nature of this study, a large number of patients with portal thrombosis were excluded for its high prevalence in patients with PH, which might lead to potential selection bias and may impair the reproducibility of the results. Second, the sample size of patients for follow-up and those with disease progression was limited, and the interpretation of the results should be carried out with caution and still needs further validation with a large-scale sample size. Third, the findings of this preliminary study are less than ideal, which is the main limitation of this research. We are now collecting more eligible patients, and we plan to conduct consecutive research with a large sample size to improve the predictive performance. We hope that the preliminary results could be suggestive for researchers. Finally, this study is retrospective and was carried out in a single-center institution, and more prospective multicenter investigations are needed to better validate the results in clinical practice.

In conclusion, our study demonstrated that CT-based texture signatures from the liver or spleen may have the potential to stratify PH and predict patient survival. The results still need to be corroborated by further multicenter prospective studies.

## Data Availability Statement

The original contributions presented in the study are included in the article/Supplementary Material, further inquiries can be directed to the corresponding author/s.

## Ethics Statement

The studies involving human participants were reviewed and approved by the West China Hospital Ethics Committee. The patients/participants provided their written informed consent to participate in this study.

## Author Contributions

SW and YW contributed to the conception and design of the study. SW and CY organized the database. XZ carried out data statistics and analysis. SW wrote the manuscript. FH and BS revised the manuscript. All authors have read and approved the final manuscript.

## Conflict of Interest

XZ was employed by the company Pharmaceutical Diagnostics, GE Healthcare. The remaining authors declare that the research was conducted in the absence of any commercial or financial relationships that could be construed as a potential conflict of interest.

## Publisher’s Note

All claims expressed in this article are solely those of the authors and do not necessarily represent those of their affiliated organizations, or those of the publisher, the editors and the reviewers. Any product that may be evaluated in this article, or claim that may be made by its manufacturer, is not guaranteed or endorsed by the publisher.

## Abbreviations

PH: portal hypertension
PVP: portal vein pressure
HVPG: hepatic venous pressure gradient
CSPH: clinically significant portal hypertension
HE: hepatic encephalopathy
HCC: hepatocellular carcinoma
LS: liver stiffness
TE: transient elastography
CT: computed tomography
TIPS: transjugular intrahepatic portosystemic shunt
PFS: progression-free survival
FHVP: free hepatic venous pressure
WHVP: wedged hepatic venous pressure
ROIs: regions of interest
GLCM: gray-level cooccurrence matrix
GLSZM: gray-level size zone matrix
GLRLM: gray-level run length matrix
NGTDM: neighboring gray-tone difference matrix
GLDM: gray-level dependence matrix
ICC: intraclass correlation coefficient
ROC: receiver operating characteristic
AUC: curve and the area under the curve
INR: international normalized ratio
OR: odds ratio
SD: standard deviation
IQR: interquartile range
HR: hazard ratio
PT: prothrombin time
CTP: Child-Turcotte-Pugh.
