# Supplementary material for: Computed Tomography-Based Texture Features for the Risk Stratification of Portal Hypertension and Prediction of Survival in Patients With Cirrhosis: A Preliminary Study
Source: Front Med (Lausanne). 2022 Apr 1;9:863596. doi: 10.3389/fmed.2022.863596 (PMC9010529; doi:10.3389/fmed.2022.863596)
Supplement: Supplementary file 1 [file Data_Sheet_1.docx]

**Supplementary Table 1** Univariate Cox proportional hazard regression of clinical characteristics for survival.

| **Variables** | **Survival analysis for PFS** | |
| --- | --- | --- |
|  | **HR (95% CI)** | ***P*** |
| Age | 1.03 (0.98-1.09) | 0.233 |
| Gender | 0.83 (0.23-2.97) | 0.767 |
| Etiology of cirrhosis | 1.02 (0.62-1.68) | 0.947 |
| EVB history | 1.71 (0.22-13.17) | 0.578 |
| Ascites | 0.71 (0.20-2.59) | 0.620 |
| Hypersplenism | 3.80 (1.31-10.99) | 0.021 |
| HE | 4.27 (1.33-13.64) | 0.031 |
| TBIL | 1.00 (0.97-1.04) | 0.805 |
| Albumin | 0.93 (0.85-1.00) | 0.064 |
| Globulin | 1.05 (0.98-1.12) | 0.180 |
| ALT | 0.99 (0.98-1.01) | 0.885 |
| AST | 1.00 (0.99-1.01) | 0.544 |
| INR | 1.72 (1.07-2.77) | 0.077 |
| PLT | 0.99 (0.98-1.01) | 0.411 |

HR, hazard ratio; CI: confidence interval; PFS, progression-free survival; EVB, esophageal variceal bleeding; HE: Hepatic encephalopathy; ALT, alanine aminotransferase; AST, aspartate aminotransferase; INR, international normalized ratio; PLT, platelet count.

**Supplementary Figure 1**


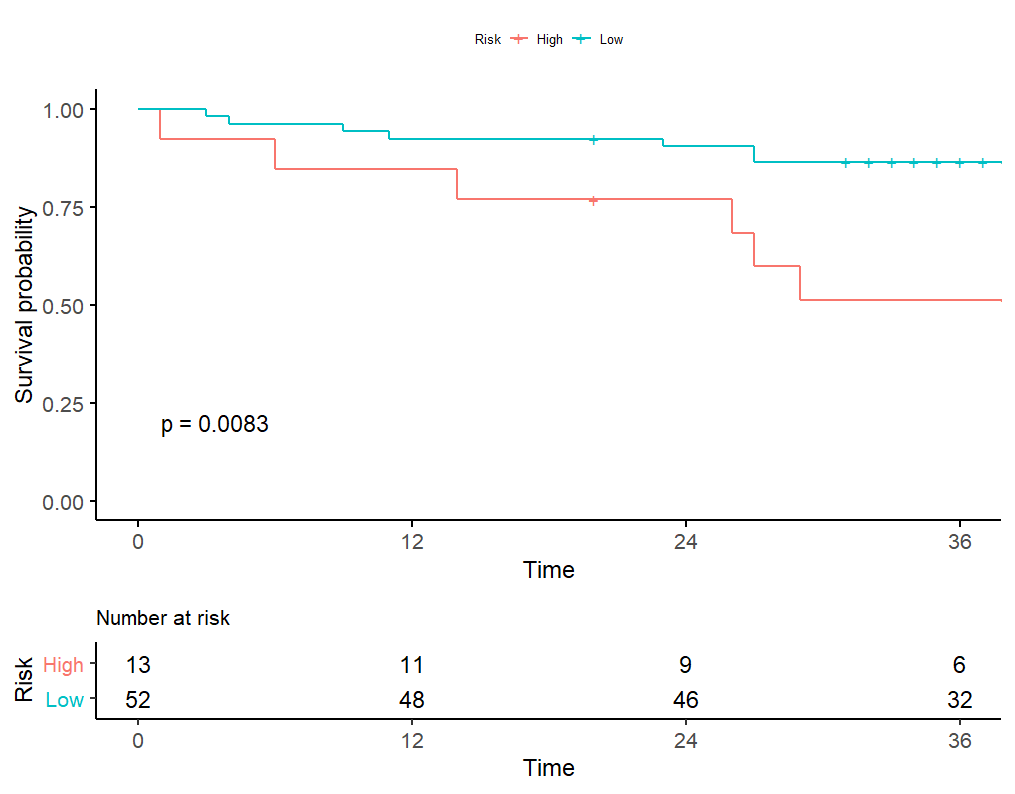


**Supplementary Figure 1.** Kaplan–Meier curve of clinical variable of hypersplenism for the evaluation of patient survival. As the picture depicts, this biomarker could stratify patient with portal hypertension into high- or low-risk groups (log-rank test, *p* < 0.05).

**Supplementary Figure 2**
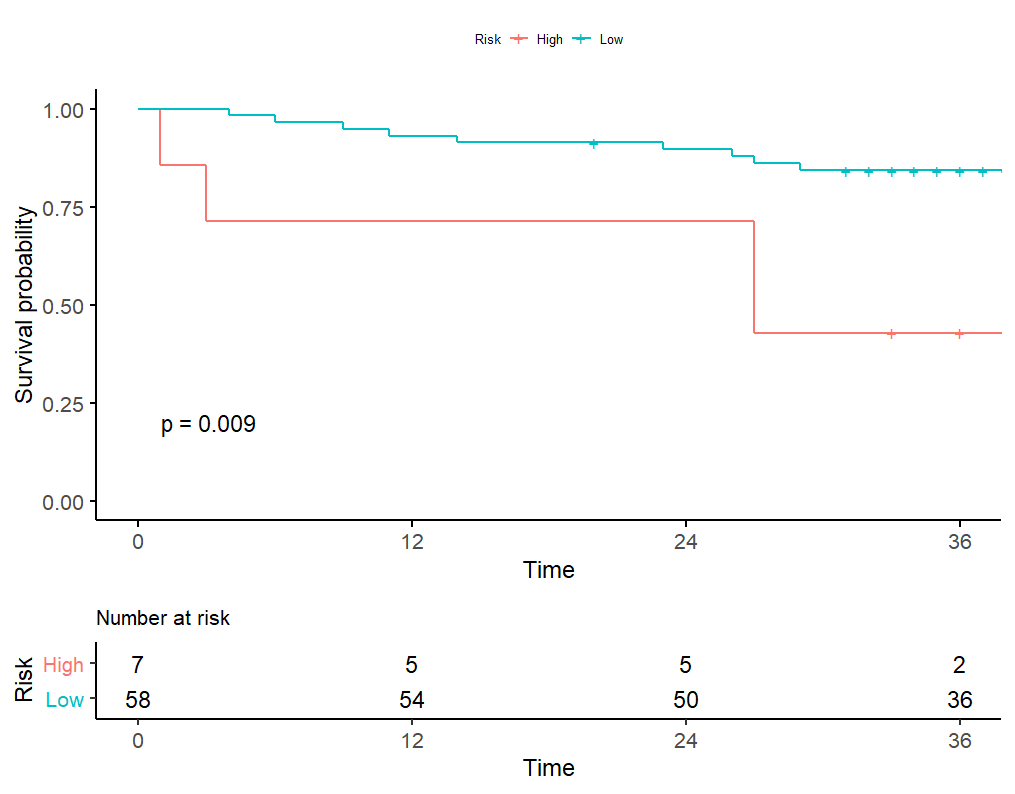


**Supplementary Figure 2.** Kaplan–Meier curve of clinical variable of hepatic encephalopathy for the evaluation of patient survival. As the picture depicts, this biomarker could stratify patient with portal hypertension into high- or low-risk groups (log-rank test, *p* < 0.05).
